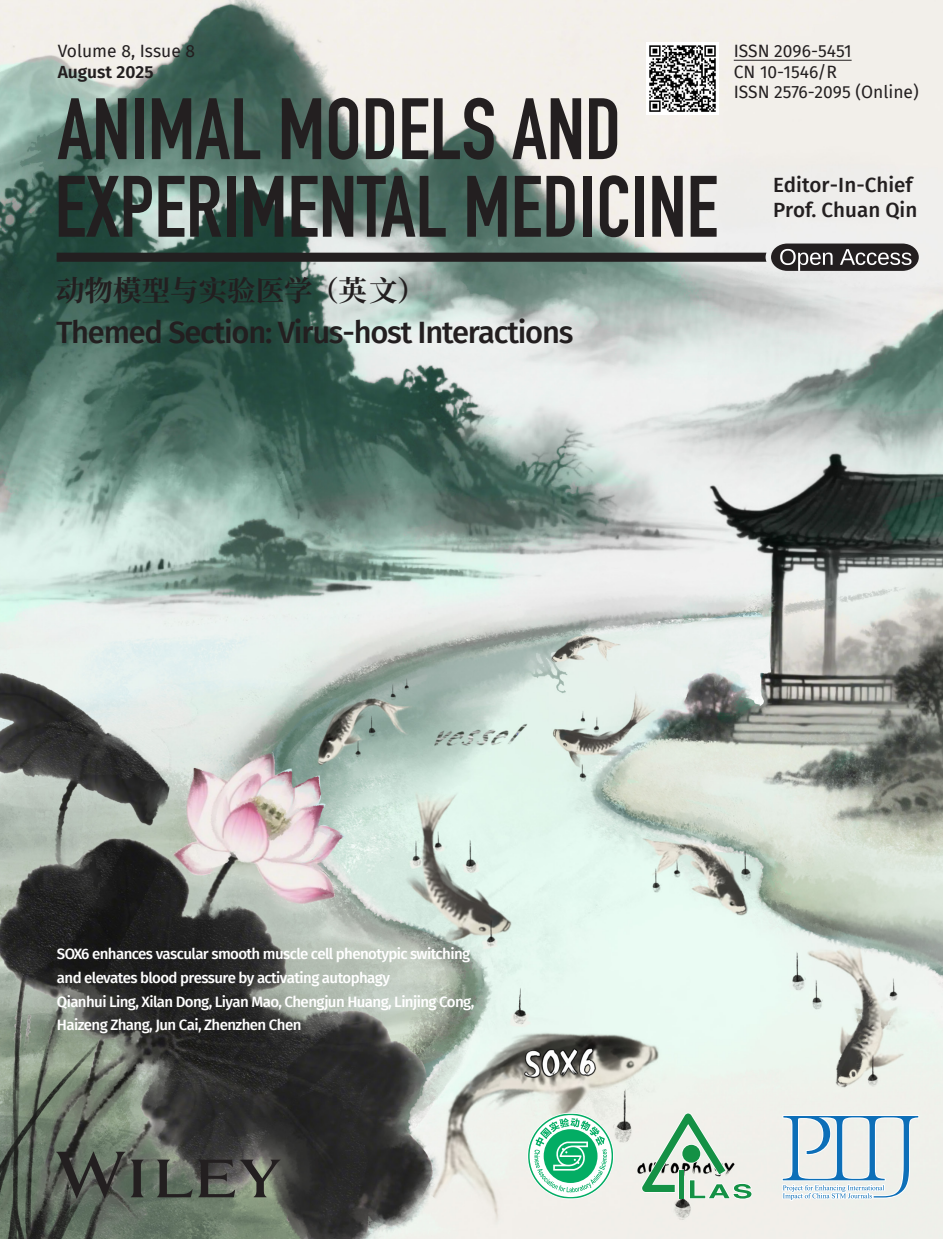# Cover Picture

**DOI:** 10.1002/ame2.12539

**Published:** 2025-09-26

## Abstract

This cover image is based on the article “SOX6 enhances vascular smooth muscle cell phenotypic switching and elevates blood pressure by activating autophagy” reported by Qianhui Ling, Xilan Dong, Liyan Mao, Chengjun Huang, Linjing Cong, Haizeng Zhang, Jun Cai, Zhenzhen Chen. (https://doi.org/10.1002/ame2.70046). The artwork depicts the entire organism as a vibrant landscape, with blood vessels pictured as rivers meandering through the body, showcasing their fundamental role in physiology. At the core of this metaphor, SOX6 proteins swim through these vascular waterways like graceful fish, dynamically regulating vascular function and maintaining physiological homeostasis by feeding on the “bait” of autophagy–a cellular process essential for their regulatory activity. This visual narrative not only illustrates the study's core findings but also highlights the concept that the human body is an integrated organic whole.